# An online analytical processing multi-dimensional data warehouse for malaria data

**DOI:** 10.1093/database/bax073

**Published:** 2017-10-07

**Authors:** S M Niaz Arifin, Gregory R Madey, Alexander Vyushkov, Benoit Raybaud, Thomas R Burkot, Frank H Collins

**Affiliations:** 1 Department of Computer Science and Engineering, University of Notre Dame, Notre Dame, Indiana, USA; 2 Center for Research Computing, University of Notre Dame, Notre Dame, Indiana, USA; 3 Institute for Disease Modeling, Bellevue, Washington, USA; 4 Australian Institute of Tropical Health and Medicine, James Cook University, Cairns, Queensland, Australia; 5 Department of Biological Sciences, University of Notre Dame, Notre Dame, Indiana, USA

## Abstract

Malaria is a vector-borne disease that contributes substantially to the global burden of morbidity and mortality. The management of malaria-related data from heterogeneous, autonomous, and distributed data sources poses unique challenges and requirements. Although online data storage systems exist that address specific malaria-related issues, a globally integrated online resource to address different aspects of the disease does not exist. In this article, we describe the design, implementation, and applications of a multi-dimensional, online analytical processing data warehouse, named the VecNet Data Warehouse (*VecNet-DW*). It is the first online, globally-integrated platform that provides efficient search, retrieval and visualization of historical, predictive, and static malaria-related data, organized in data marts. Historical and static data are modelled using star schemas, while predictive data are modelled using a snowflake schema. The major goals, characteristics, and components of the DW are described along with its data taxonomy and ontology, the external data storage systems and the logical modelling and physical design phases. Results are presented as screenshots of a Dimensional Data browser, a Lookup Tables browser, and a Results Viewer interface. The power of the DW emerges from integrated querying of the different data marts and structuring those queries to the desired dimensions, enabling users to search, view, analyse, and store large volumes of aggregated data, and responding better to the increasing demands of users.

**Database URL:**

https://dw.vecnet.org/datawarehouse/

## Introduction

Malaria is probably the third most important pathogen-specific cause of human morbidity and mortality in the world today. The disease is caused by parasites of the genus *Plasmodium*, which are transmitted between humans by *Anopheles* mosquitoes. The populations of sub-Saharan Africa experience the highest burden of the disease with most of the approximately 627 000 annual deaths (1).

Malaria-related data management shares many challenges faced by common generic biological data management tasks. The complex nature of the disease and the integration of data from heterogeneous, autonomous, distributed, and fast-changing data sources make the task especially challenging. In this article, we describe the design and implementation of a multi-dimensional, online analytical processing (OLAP) data warehouse (DW), named *VecNet-DW* (2), for malaria-related data. *VecNet-DW* uses open-source software modules and frameworks integrated in the Vector-Borne Disease Network (VecNet) (3). VecNet is an online portal which facilitates access to mathematical/simulation models and the data to parameterize these models. It allows users to understand how different vector control and parasite-targeted interventions affect malaria transmission in different geographical areas where heterogeneity in vector behaviours can dramatically impact intervention effectiveness. Such analyses will identify where new strategies to eliminate malaria are needed. Hence, VecNet enables its users to collaboratively conduct research and to analyse and share results from multiple malaria models. In this article, we discuss the major goals, characteristics, and components of *VecNet-DW*, along with its data taxonomy and ontology, the external data storage systems, and the logical modelling and physical design phases. 

### Data management challenges for malaria-related data

The complex epidemiology of malaria presents several data management challenges involving (i) heterogeneity in data contents, and (ii) integration of data stored in different formats. For example, the World Malaria Report (WMR) (4) stores data on recommended policies and strategies as well as intervention coverages estimates while the Malaria Atlas Project (5) stores geographical maps on dominant malaria vectors, estimated human populations at risk, etc., the Pacific Rainfall Database [PacRain, (6)] and the Global Surface Summary of Day [GSOD, (7)] store weather-related malaria data, and so on.

Often, these data are stored in different formats [e.g. relational databases, spreadsheets, comma-separated values (CSV), plain text files and others], sometimes with an unclear understanding of its meaning, forcing the user to decipher the definition of the data content from other supporting and/or accompanying information (metadata) provided with the original data. For example, both PacRain (6) and GSOD (7) provide additional information in separate files that describe the meaning of content, column headers, codes used in the content etc. with publishers changing the data definitions or columns, augmenting new types and entries or reordering existing data, requiring users to learn the changes to understand and use the data.

In recent years, the need to integrate heterogeneous malaria-related data has grown. Increasingly, storage of data in flat files (e.g. in spreadsheets or CSV files) or traditional relational database management systems (RDBMSs) cannot cope with the growing demand of this integration process. The primary concern of traditional databases is to ensure concurrent multi-user access, data consistency, recovery techniques and the day-to-day operations of an organization (8). However, due to their highly normalized nature, they cannot always satisfy the complex requirements of advanced data analysis and often perform poorly when executing complex queries or aggregating large volumes of data (8, 9).

Any comprehensive digital framework dealing with malaria data such as VecNet (3) must incorporate new data sources while maintaining the consistency and quality of the existing content when the content from the ‘same’ data sources changes over time. A well-designed DW, based on dimensional modelling (DM) techniques, may eliminate some of these challenges, and respond better to the increasing needs of users (8–10). Some of the other major differences between traditional databases and DWs follow:

- **Nature of operations (queries):** users of a traditional database usually deal with one record at a time, and repeatedly perform the same operational tasks (8–10); users of a DW almost never deal with one record at a time; rather, they are mostly interested in aggregates of data/contents that often represent hundreds or thousands of records which are searched and compressed into an answer set (10, 11); hence, DW users usually have drastically different needs than traditional database users.
- **Data sharing:** each traditional database often exists as a natural isolated application, with little investment made to sharing common data (e.g. date, location etc.) with other databases in the organization. In a DW, these databases are re-engineered to ensure consistency (11).

DWs use a special design technique known as DM, which views data as consisting of facts linked to several dimensions of interest (8, 11). Being different from the more traditional entity-relationship modelling (ER) technique, DM focuses on aggregations, which are performed to summarize many records with potential filtering based on attributes of relevant facts and dimensions (11).

Several bioinformatics- and life science-focused data storage systems (databases and DW) exist to support the data needs of various diseases (12–23). However, a globally-integrated online resource to exclusively address different aspects of malaria-related data, which may serve as the equivalent of a malaria decision support system, does not exist. For malaria, it can serve both long-term strategic or short-term tactical decision-making. For example, based on existing malaria control interventions, it can help in making decisions about introducing new, novel interventions in a particular location by analysing the long-term demographic, entomological, epidemiological, and other characteristics of that location (e.g. the incidence and prevalence data collected and stored over several decades). As an example of short-term decision making, a DW can help predict the outcome of modifying and/or increasing the levels of coverage for existing vector control interventions, or introducing new drugs or insecticides, for which the immediate impact needs to be measured.

### *VecNet-DW*: the VecNet data warehouse

*VecNet-DW* is an online multi-dimensional DW, designed specifically to store, access, and analyse malaria-related data scattered in heterogeneous forms across many data storage systems. Although there are other online data storage systems that provide malaria-related data, such as the EuPathDB Bioinformatics Resource Center (23) which provides genomic-scale datasets associated with eukaryotic microbes, or the Malaria Atlas Project (5) which provides geographical data on dominant malaria vectors, a globally integrated online resource for the storage and analysis of many different aspects of the disease does not exist. *VecNet-DW* is the first integrated online platform designed exclusively for providing OLAP functionalities (slice and dice, roll up and roll down etc.) as well as efficient search, retrieval, analysis, and visualization of historical, predictive/synthetic, and static malaria-related data. Its DM intuitively views the multi-dimensional data as data cubes. The four prime characteristics of *VecNet-DW* are:

- **Subject-oriented:***VecNet-DW* targets multiple subjects of analysis according to the requirements of malaria decision-making managers/users at various levels; these subjects include data on existing and new malaria control interventions, household surveys, antimalarial drugs, entomological inoculation rates (EIRs), distributions and abundances of dominant vector species, various models and studies (e.g. mathematical, agent-based, field-based), model-specific parameters etc.
- **Integrated:** the contents of *VecNet-DW* include integration of data from various external data storage systems, historic archives, or operational source systems (OSSs); some of these OSSs include WMR (4), GSOD (7) etc.
- **Nonvolatile:** once ingested, *VecNet-DW* data are read-only and persistent with modification/curation of content from the OSSs only in the data staging area (described later); thus, *VecNet-DW* stores curated and nonvolatile data, retained for long-term analysis and aggregation purposes.
- **Time-varying:***VecNet-DW* records the temporal evolution of data from the OSSs for a long period of time (typically many years); for example, it may keep track of temporal malaria data on endemicity, incidence, prevalence, etc. ranging over several decades.

**Figure 1.**
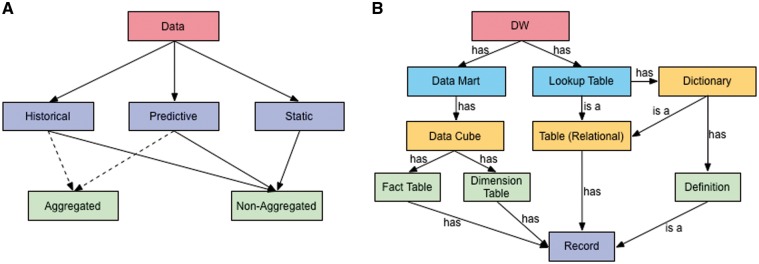
Data taxonomy and ontology. **(A)** The taxonomy organizes *VecNet-DW* data into three broad categories: (i) historical data, which may range over several decades, are collected, modelled and stored from OSSs and the literature; (ii) predictive (or synthetic) data, which are mostly generated as outputs of different types of malaria models; and (iii) static data, which are mostly non-numeric (textual), also collected and modelled from the OSSs and the literature, and stored in the lookup tables. Both the historical and predictive categories may encompass aggregated and non-aggregated forms, while the static category may only encompass the non-aggregated form. **(B)** An ontology for VecNet-DW. Entities and their relationships are represented by rectangles and labelled arrows, respectively. Entities within the same ontology level are marked with the same colours. The root level (Level 1) has a single entity as the DW, VecNet-DW, which has multiple data marts and lookup tables as Level 2 entities. Each data mart can be modelled as one or more data cubes (Level 3). Each data cube usually has one or more fact tables (Level 4), and multiple dimension tables (Level 4), all of which store records (facts and dimensions, Level 5) of varying granularities. Each lookup table can be either a relational table or a dictionary (Level 3). A relational table may contain records that are mostly dimension-less. A dictionary may in turn be a relational table, or may contain semantic definitions (Level 4), which are stored as records.

*VecNet-DW* serves a wide range of (often overlapping) user groups, including researchers, modellers, malaria control managers, product developers, etc. It stores three broad categories of data: historical, predictive, and static. The interrelations between these categories are crucial because predictions and interpretations of biological data are often made by comparing predictive data against existing historical data. Also, in some cases, both of these categories may interrelate to the static category. Both the historical and predictive categories may encompass aggregated and non-aggregated storage forms, while the static category may only encompass the non-aggregated form. A taxonomy defining groups of data stored in *VecNet-DW* on the basis of shared characteristics, is shown in Figure 1A.

‘Historical data’, possibly ranging over several decades, needs to be stored so that users can analyse malaria trends and/or assess the impact of malaria control interventions by considering various aspects of the data (e.g. demographic, entomological, epidemiological etc.). On the other hand, ‘predictive’ or ‘synthetic’ data, mostly generated as outputs of various malaria models, is useful because they can simulate specific situations with precise settings to understanding past trends and behaviours, leading to discovery of new insights about the current environment. Predictive data allows users to pose ‘what-if’ questions to assess the outcome of various strategies, rather than being limited to qualitative decision models. In VecNet, since multiple individual models will be run (possibly with distinct but overlapping sets of input parameters), and produce intermediate and final outputs in various formats, model-specific input/output must also be considered in designing the DW to store the predictive data. Predictive data may traverse the ‘past’ and the ‘future’. The ‘past’ data allow users to fill in missing historical data, to compare outputs from the stochastic models, and to validate the models against real, measured, or otherwise verified data (often originating from field-based studies). The ‘future’ data, generated by prediction-based models, may inform decision makers at different levels of malaria control and eradication campaigns. For example, public health officials may make decisions on purchasing and distributing malaria control products (such as bed nets, drugs, diagnostic tests and insecticides) using anticipated needs predicted by the models. Since not all vector control interventions are equally efficient in different regions of the world, the ‘future’ data, based upon the stochastic modelling for specific locations, may also prove helpful to prioritize, schedule, manage, and assess the impact of finite resource allocations based on analyses of several scenarios with multiple variables simulated simultaneously. Last, ‘static’ data, which are mostly non-numeric (textual), are also collected and modelled from the OSSs and the literature, and stored in the lookup tables (described later).

A simple ontology for *VecNet-DW* is presented in Figure 1B. It depicts the high-level description of concepts and their relationships in the upper parts of *VecNet-DW* knowledge domain, using a representative vocabulary (described in Supplementary Material S1). The DW (*VecNet-DW*) exists as the single entity in the root level (Level 1), and has multiple data marts and lookup tables as Level 2 entities. Each data mart can be modelled as one or more data cubes (Level 3). Each data cube usually has one or more fact tables (Level 4), and multiple dimension tables (Level 4), all of which store records (facts and dimensions, Level 5) of varying granularities. Each lookup table can be either a relational table or a dictionary (Level 3). A relational table may contain records, which are mostly dimension-less. A dictionary may in turn be a relational table, or may contain semantic definitions (Level 4), which are stored as records.

## Materials and methods

In this section, the modelling process of the three categories of data supported by *VecNet-DW* is described, together with the major components, the data cubes and OLAP operations, and the implementation frameworks. The conventions used in this article for different types of entities are described in Table 1.

**Table 1.** Conventions used in this article for different types of entities

Entities	Text style	Letter case	Color code	Examples
Dimensions, dimension tables	italic, always singular	all lowercase	brown	‘date’, ‘location’
Definitions	italic, always singular	sentence case	N/A	‘Human blood index’, ‘Sugar meal frequency’
Facts	regular	sentence case	N/A	number of long-lasting insecticidal nets, % ITN coverage
Fact tables, data marts	regular, always plural	title case	light blue	Demographics, Household Surveys, Weather
Lookup tables	regular, always plural	title case	N/A	Species Bionomics, Entomological Endpoints

N/A means not applicable. The color code is followed in figures and supplementary files. Conformed dimensions ‘location’ and ‘date’, when appropriate, are marked in different colors, and mentioned in the text.

### Data sources

The data sources ingested in the data marts and the lookup tables in *VecNet-DW* are available in the *VecNet* Digital Library (24), which is another major component of *VecNet*. The *VecNet* Digital Library also contains related links/URLs to the original data sources and other metadata.

Table 2 describes all datasets, including data sources, release dates, file formats, and URLs in the *VecNet* Digital Library from which each dataset can be downloaded.

**Table 2.** Description of datasets ingested in data marts and lookup tables in *VecNet-DW*

Name	Description	Data source	Release date	File format	References
Demographics	Population data stratified by age groups	United Nations: Desa/Population Division	2010	Microsoft Excel	(25)
Gross National Income	Gross national income of a country	The World Bank	2012	Microsoft Excel	(26)
Household Surveys	Household surveys of mosquito net ownership and usage	World Health Organization (WHO)	2011	Microsoft Excel	(27)
Households	Number of households and life expectancy by country	World Malaria Report published by the WHO	2013	Microsoft Excel	(28)
Interventions	Operational coverage data for interventions ACT, IRS, and ITN	World Malaria Report published by the World WHO	2012, 2015	Microsoft Excel	(29, 30)
Weather	Daily weather data	National Climatic Data Center, National Oceanic and Atmospheric Administration	2011	CSV, PDF	(31)
Species Bionomics	Expert opinion lookup table covering Siaya District in Nyanza Province of Western Kenya	VecNet Partner/Expert John E Gimnig, Tanya Russell, Frank H Collins	2012	Microsoft Excel	(32, 33)
Entomological Parameters	Measurable entomological endpoints impacted by vector control paradigms.	VecNet Partner/Expert Robert Farlow	2012	Microsoft Excel	(34)
Intervention Coverage in Africa	IRS and ITN Coverage in Africa based on demographic, health survey, etc.	World Malaria Report published by the WHO	2012, 2015	Microsoft Excel	(29, 30)
Representative Workflow Parameters, Vector Species Parameters, and Vector Species Sensitivity Parameters	Synthetic data to programmatically generate input files for VecNet’s stored EMOD (37) model. Data are based on the Species Bionomics table, and model-specific parameters are determined using calibration.	VecNet Partner/Expert John E Gimnig, Tanya Russell, Frank H Collins	2012	JSON, Plain text, CSV	(35)

### Modelling historical data

**Figure 2.**
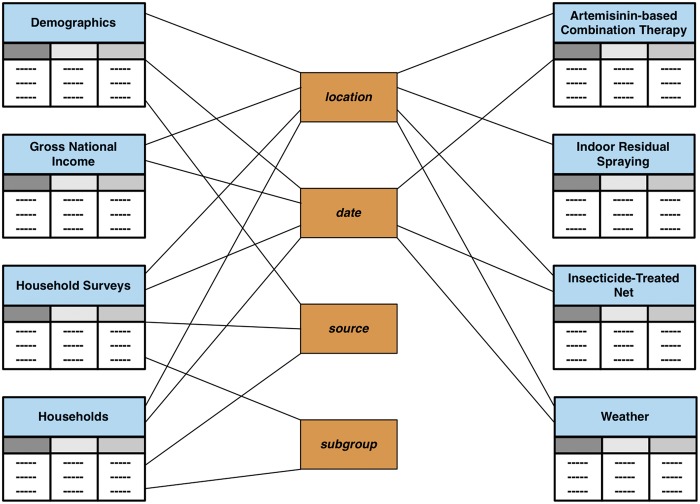
Constellation schema for historical data. The constellation schema, also known as the galaxy schema, is a collection of simple star schemas. It ties together all the fact tables and dimension tables contained within all data marts representing historical data. The connections link fact tables to corresponding dimension tables. The fact tables are (partially) shaded in light blue. Dimensions are shaded in brown.

In *VecNet-DW*, historical data are modelled using a constellation schema, which is a collection of multiple star schemas. It ties together all the fact tables and dimension tables, contained within the data marts that represent historical data. Historical data are ingested into *VecNet-DW* from external data storage systems, sometimes also known as OSSs. The constellation schema, shown in Figure 2, includes high-level views of all OSSs. Note that although we could combine multiple data marts (and the corresponding ingested data originating from multiple OSSs), most of the data marts are segregated in anticipation of large volumes of data to be integrated in the future. Currently modelled datasets are described below.

- Demographics: describes population data stratified according to different age groups; associated with dimensions ‘location’, ‘date’ and ‘source’.
- Gross national income: describes the gross national income (total domestic and foreign output claimed by residents) of a country; associated with dimensions location and date.
- Household Surveys: describes household surveys of mosquito net ownership and usage data, as part of the WMR 2010 (4); associated with dimensions location, date, source and subgroup.
- Households: describes the number of households in a country; associated with dimensions location, date, source and subgroup.
- Interventions: contains operational coverage data for three malaria control interventions: artemisinin combination therapy (ACT), indoor residual spraying (IRS) and insecticide-treated net (ITN) (4), which are stored as individual fact tables; associated with dimensions ‘location’ and ‘date’.
- Weather: contains a collection of daily weather data from GSOD (7); associated with dimensions ‘station’ and ‘date’.

### Modelling static data

The static data stored in *VecNet-DW* currently entails the following lookup tables:

- Species bionomics: specifies vector species bionomics parameter values.
- Entomological parameters: outlines major measurable entomological endpoints impacted by vector control paradigms.
- ITN coverage in Africa: describes ITN coverage in Africa based on demographic, health survey and WMR data.
- IRS coverage in Africa: describes IRS coverage in Africa based on demographic, health survey and WMR data.
- Representative workflow parameters: describes vector species parameters to calibrate models in VecNet’s Transmission Simulator interface (36).
- **Vector species parameters:** specifies vector species parameters for transmission models.
- **Vector species sensitivity parameters:** specifies additional vector species parameters for transmission models.

### Modelling predictive data

‘Predictive’ (or ‘synthetic’) data are mostly generated as outputs of different types of malaria models. Due to the need of users’ (mostly researchers) privacy protection, predictive data are marked as ‘private’, and only a selected subset is made available for public viewing via another interface (the Transmission Simulator under the *VecNet* portal, 36).

**Figure 3.**
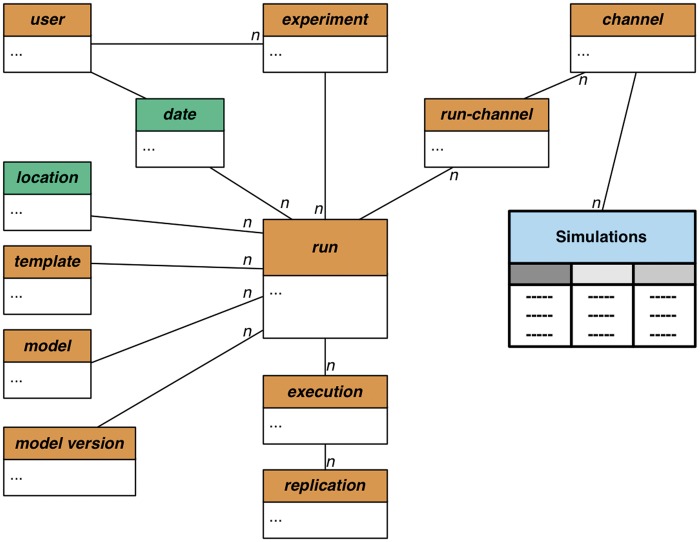
Snowflake schema for predictive data. The snowflake schema for predictive data consists of the dimension tables and one single fact table, Simulations (shaded in light blue). It contains time series data as direct outputs by the stored models (EMOD and OpenMalaria), and is connected to several dimensions (shaded in light brown). The ‘run’ dimension is snowflaked: a run represents a specific simulation run of a particular model, with the current version stored in model version. Its spatio-temporal information is stored via the conformed dimensions ‘location’ and ‘date’ (shaded in green), respectively. Each simulation run is also associated with a ‘template’, which describes all simulation parameters, along with their respective values. The output of a simulation run is stored in multiple ‘channels’. A user submits a simulation job as an ‘experiment’. Each experiment is transformed into runs, which, in turn, are broken into ‘executions’. An execution thus represents a single realization of the run configuration. To allow replications of a simulation run, the ‘execution’ dimension is coupled with the ‘replication’ dimension. The symbol *n* represents the many side of a one-to-many relationship.

*VecNet-DW* independently stores the simulation outputs from two models, namely EMOD (37) and OpenMalaria (38), using a snowflake schema (a normalized version of a star schema) as shown in Figure 3. This approach provides flexibility and extensibility, since it allows a model’s outputs to be seamlessly integrated. Currently, most of the simulation outputs assume the form of time series data. The snowflake schema consists of several dimensions and one single fact table, Simulations, which contains time series data collected as outputs from stored models. It is connected to several dimension tables including ‘run’, ‘replication’, ‘channel’, ‘model’, ‘model version’ etc. A run represents a specific simulation run of a particular model (with the current stored model version). Its spatio-temporal information is stored via the conformed dimensions ‘location’ and ‘date’. Each simulation run is also associated with a ‘template’, which typically refers to a description of configurations (in either hierarchical or flattened form). The templates describe all parameters, along with their respective values, which are to be used by simulations with baselines, campaigns (including interventions) or other configuration settings.

The output of a simulation run is stored in multiple channels. Each ‘channel’ represents a specific type of output (e.g. adult mosquito population, human biting rate, parasite prevalence etc.), which is accumulated once per simulated time step and written to output files. Depending on the type of analysis, the channels may vary between models, and also within the same model. For all channels, the time steps and their corresponding output values are individually stored, which allows the DW to generate aggregates over any time range within a channel for any given run. A ‘user’ submits simulation jobs as experiments. Each ‘experiment’ is transformed into runs, which, in turn, are broken into executions. An ‘execution’ thus represents a single realization of the run configuration, coupled with a selected ‘template’ and associated with a stored ‘model’. The primary goal of transforming a run into multiple executions is to allow parameter sweeps of a particular configuration, by allowing each execution to represent a single configuration in which an individual value of the desired parameter, selected from a specified range, can be submitted.

To allow replications of a simulation run, the execution dimension is also coupled with the ‘replication’ dimension. Since the stored models involve substantial stochasticity in the forms of probability-based distributions, equations etc., performing sufficient number of replicated runs (with identical parameter settings) is important for validation of the results. In addition, to increase the performance of the DW, search indexes are built on the fact table (Simulations) for selected dimensions (‘run’, ‘replication’ 'and ‘channel’). The snowflake schema thus allows for efficient search and aggregation of the simulation output data, enabling *VecNet-DW* to answer a variety of research questions.

### Design

#### DW components

**Figure 4.**
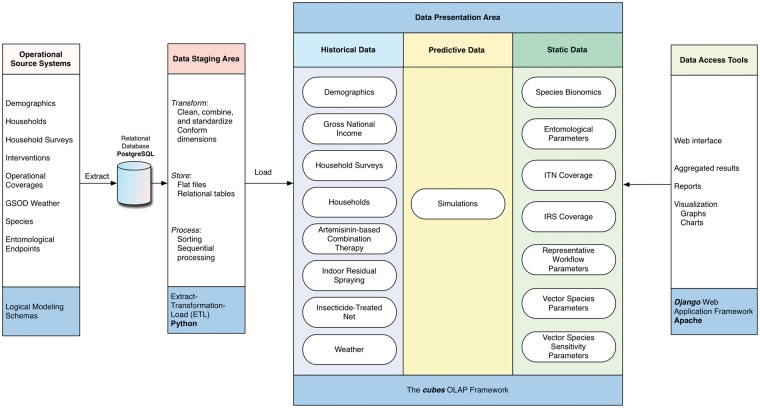
*VecNet-DW* components. The four separate and distinct components of the DW environment: operational source systems, data staging area, data presentation area and data access tools. Each component serves specific functions, as described in Methods. The modelling phases and/or implementation technologies used are listed at the bottom in the blue-shaded boxes for all components.

The four distinct components of *VecNet-DW* are shown in Figure 4, and are described below:

- **OSSs** **:** The OSSs are external data storage systems (mostly flat files) whose contents are ingested into *VecNet-DW*. After transferring the content of each OSS, we identify the dimensions and facts, and then decide on existing or new data marts to ingest the content. In this logical modelling phase, we pay particular attention to identify the correspondence between the data marts and the ‘conformed’ dimensions, and populate the bus matrix (see Supplementary Material S2). At the end of this phase, as each data mart have been logically modelled, the constellation schemas tie together all the fact tables, dimension tables, lookup tables, and dictionaries. As we perform the domain analysis for individual data marts, we analyse the contents and data type of each attribute in the OSSs’ source files. To ensure that each record can be uniquely identified within the data source (i.e. the referential integrity), we determine and assign primary keys for all dimensions and facts (if no such keys can be found, we assign additional candidate keys).
- **Data staging area:** Once the content of each OSS has been logically modelled, we perform extract-transformation-load (ETL) processes on the content. We extract the content by reading and understanding the source data, and perform data transformations by data cleansing (done primarily by correcting misspellings, dealing with missing or null elements, and parsing into standard formats), and assign DW keys. Then, following the logical model and schemas, we load the content into a Postgre structured query language (SQL) RDBMS (39) by using a Python SQL toolkit named SQLAlchemy (40). To adhere to the key architectural requirements for the data staging area, we keep the PostgreSQL instance off-limits to our users, and isolate it from our query and presentation services.
- **Data presentation area:** In the data presentation area, following the constellation schemas, we organize and store the most detailed (i.e. ‘atomic’) data from the PostgreSQL RDBMS into an open-source OLAP Python framework named ‘cubes’ (41).
- **Data access tools:** Currently, the data access tools include the open access, Web-based graphical user interface front-end developed in Django (42), which allows users to have unrestricted access via the published interface (2). Aggregation operations for selected data marts are also supported.

#### Data cubes and OLAP operations

The DM of *VecNet-DW* views the data stored in the DW as cubes of data. Each data mart can be modelled with one or more data cubes. Data points inside a cube represent the stored facts (measurements) for specific combinations of the associated dimensions. The multidimensional cube view of data promotes understandability by allowing data visualization in a concrete and tangible way (11). A result of a multidimensional query is either a cell, a two-dimensional slice, or a multidimensional sub-cube (9). Common OLAP operations on the multidimensional data stored in *VecNet-DW* include:

- **Roll up and roll down:** these operations allow the user to navigate among levels of data ranging from the most summarized (up) level to the most detailed (down) level, along any hierarchical dimension. ‘Roll up’, also known as ‘aggregation’, or ‘drill up’, permits higher levels of summarization. ‘Roll down’, also known as ‘drill down’, is the opposite operation, which permits viewing more fine-grained levels of data. The design and granularity of the DW determines its ability to roll up or down. The summarization rule (for rolling up) may involve computing the sum, average, maximum, or minimum values along the hierarchical dimension, or applying a set of formulas, for example:
  number of bed nets=number of ITNs+number of LLINs
- **Slice and dice:** these operations allow the user to access a DW through any of its dimensions; the process generally implies a systematic reduction of DW data in any logical combination. The ‘slice’ operation selects a rectangular subset of a data cube by choosing a single value for one of its dimensions, creating a new cube with one fewer dimension. The ‘dice’ operation produces a sub-cube by selecting specific values of multiple dimensions.

**Figure 5.**
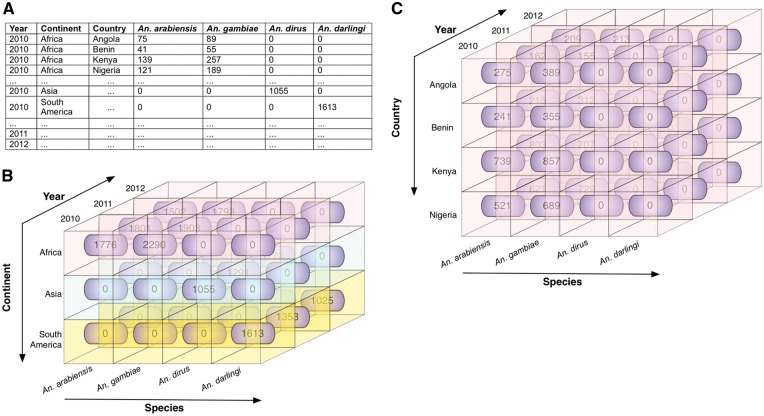
Illustrative example of roll up and roll down. These operations allow the user to navigate among levels of data ranging from the most summarized (up) level to the most detailed (down) level, along a specific dimension. **(A)** Illustrative data showing mosquito abundances for various locations (continents and countries), years, and Anopheles species. **(B)** The data cube, derived from the data in A, shows the mosquito abundance facts (numbers in rounded rectangles). The cube is associated with three dimensions: species, year, and continent, which are displayed along the three axes, with data labels coming from A. Fact cells with different values of the continent dimension are distinctly colour-coded for ease of visualization. **(C)** The roll down operation produces a more detailed view of data by rolling down one level along the hierarchical location (from continent to country). The mosquito abundances of continent Africa are rolled down to abundances for countries of Africa (Angola, Benin, Kenya, and Nigeria in this scenario). Note that when rolled up, the entire cube in C represents one row of data in B (in this case, the topmost row, representing Africa).

**Figure 6.**
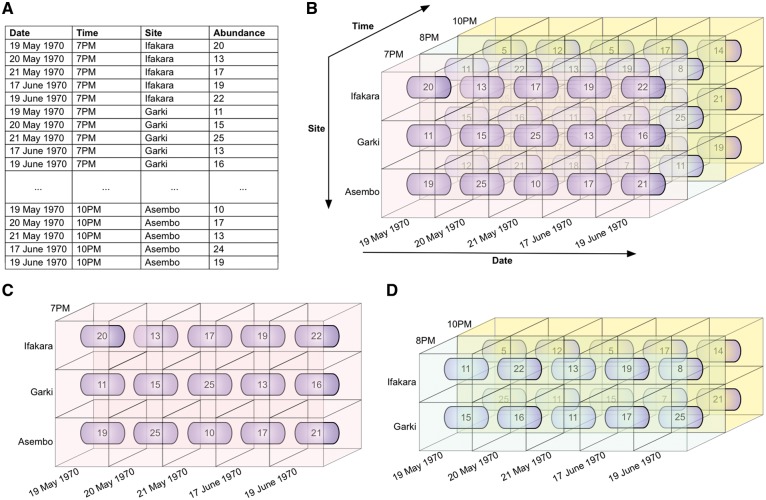
Illustrative example of slice and dice. These operations permit users to access a DW through any of its dimensions. **(A)** Illustrative data showing mosquito abundances for various dates, times, and sites (mosquito collection sites). In figures **(B–D)**, fact cells with different values of the time dimension are distinctly colour-coded for ease of visualization. (B) The data cube, derived from the data in (A). (C) The slice operation selects a rectangular subset of the cube by choosing a single value for one of its dimensions, creating a new cube with one fewer dimension. The mosquito abundances of all sites, for all dates, at 7PM are sliced out of the data cube. (D) The dice operation produces a sub-cube by selecting specific values of multiple dimensions. The abundances of sites Ifakara and Garki, for all dates, at 8 PM and 10 PM are diced out of the data cube.

These operations are illustrated in Figures 5 and 6.

### Implementation frameworks

We use ‘cubes’ (41) in the back-end to implement the DW, and Django (42) in the front-end to support the web-based user interfaces. The data flow and usage model for *VecNet-DW*, depicting the role of both frameworks, are shown in Supplementary Material S3.

#### The cubes framework

The ‘cubes’ is an open-source OLAP Python framework that uses a logical model to describe the organization, analysis, and reporting of content in a DW (41). Being independent of the physical implementation, data is described from the user’s or analyst’s perspective, emphasizing aspects of measurements (facts), aggregations, and reports. The logical model in ‘cubes’ creates an abstraction layer in the DW’s data presentation area (discussed earlier) using the JavaScript Object Notation (JSON) file format (43).

In ‘cubes’, both ‘hierarchical’ and ‘non-hierarchical’ dimensions can be shared by multiple data cubes, and thus belong to the model space. For hierarchical dimensions, the levels of the hierarchy are defined in the logical model (e.g. include ‘date’ as ‘year-month-day’ and ‘location’ as ‘continent-country-district-region’). Non-hierarchical dimensions include ‘source’, ‘subgroup’, ‘active’ ingredient, ‘chemical class’ etc. The data cubes are usually specified by a list of dimensions, facts and aggregations. It uses a set of ‘mapping rules’ to map logical attributes in the model into physical attributes in the data cube table. Each mapping rule consists of a ‘key-value’ pair, where the key refers to the attribute name of the dimension, and the value refers to the column name of the physical table.

One of the advantages of using the ‘cubes’ framework is its resilience to changes: if the metadata and the logical model are properly defined and used in an application, then most of the model changes can be handled by the application ‘without’ any modifications. This allows, e.g. adding or removing levels from a dimension without changing the DW’s reporting applications. It also supports our iterative development methodology, as we progressively advance the DW by incorporating new sources of data (see ‘Discussion’ section).

#### The Django web application framework

We use the Django Web application framework (42) in the front-end to build the Web-based user interfaces of *VecNet-DW* (2). Django is a free and open-source framework, written in Python, which encourages rapid software development and clean, pragmatic design, facilitating the tasks of creating complex, database-driven Web applications. Django is equipped with a Python-based object-relational mapper database, and also officially supports multiple databases as back-ends.

## Results

**Figure 7.**
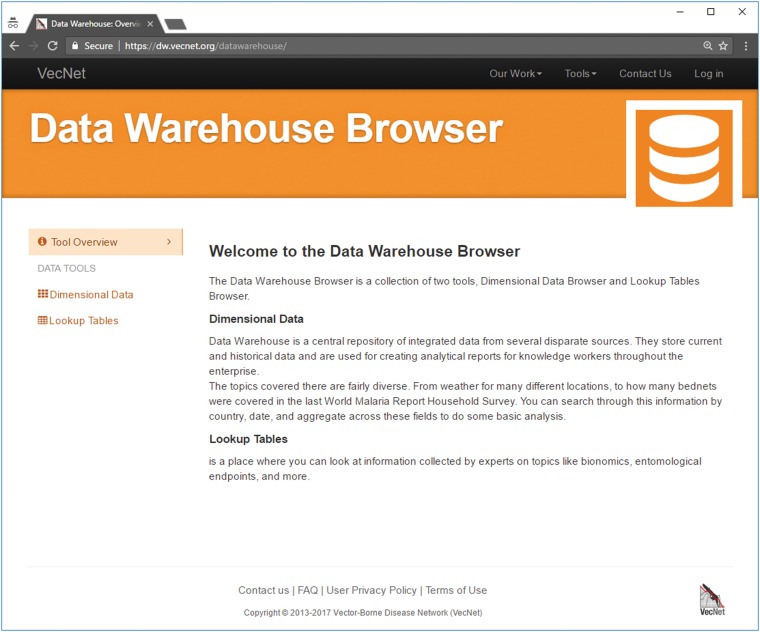
Screenshot of *VecNet-DW* homepage. The homepage provides links (on the left navigation bar) to the Dimensional Data browser and the Lookup Tables browser. The Dimensional Data browser allows users to access all data marts, which are composed of relevant facts and dimensions. The Lookup Tables browser allows users access to all lookup tables, which serve as auxiliary tables to hold static data.

The *VecNet-DW* Portal (2) consists of three major interfaces that are built according to different categories of modelled data: (i) the ‘Dimensional Data’ browser for historical data, (ii) the ‘Lookup Tables’ browser for static data and (iii) the ‘Results Viewer’ for predictive data [available for public viewing through the Results Viewer interface of the Transmission Simulator (36)]. Figure 7 shows a screenshot of *VecNet-DW* homepage, which can be accessed using modern web browsers. *VecNet-DW* homepage provides links (on the left navigation bar) to its interfaces. We discuss the interfaces in the following.

### The ‘dimensional data’ browser

The ‘Dimensional Data’ browser allows easy access to the historical data stored in *VecNet-DW*. By nature, historical data, which range over many years, are mostly numeric. Hence, aggregation over the data is important, especially for purposes of analysing trends, discovering biological insights etc., for which the non-aggregated versions are not suitable. Since it may be associated with many hierarchical as well as non-hierarchical dimensions, we employ the technique of ‘faceted search’ for browsing the data cubes. Faceted search, also known as faceted navigation or faceted browsing, is a technique for accessing information organized according to a faceted classification system, allowing users to explore information by applying multiple filters (44). For *VecNet-DW*, the filters encompass the non-hierarchical dimensions, different levels of the hierarchical dimensions, and particular values stored in these dimensions.

Using the ‘Dimensional Data’ browser, the user selects a data cube available on the left panel (see Supplementary Material S4A). This action populates the ‘Dimensions’ and ‘Measures’ (facts) panels on the bottom with associated dimensions and facts, respectively, for the selected cube. Using a ‘drag and drop’ interface, the user can then select dimensions and facts from these panels, and place (drop) them on the right under the ‘Selection Summary’ panel. Clicking the ‘Create Graph’ and ‘Create Table’ buttons (on the right panel) generates the corresponding graphs and tabular views, respectively. The resulting graphs or tabular data can then be downloaded in various formats. If there are too many items on the X-axis to display, some of them are skipped in regular intervals (axis thinning, see Supplementary Material S4B). In the following, we describe the data slicer and some faceted search examples.

#### The data slicer

The data can also be specified to slice on a given cube. As described before, the slice operation allows us to select a single value for one of the associated dimensions for a selected data cube (see Figure 6). In the ‘Dimensional Data’ browser interface, once the user drags and drops a dimension (to slice) on the Data Slicer panel, specific values for different levels within the dimension (or a single value for a non-hierarchical dimension) can be specified. Once all ‘Dimensions’, ‘Measures’ and ‘Slices’ are selected, the data can be viewed as either a graph or a table (by clicking the ‘Create Graph’ or ‘Create Table’ button, respectively). A screenshot of the Data Slicer Panel is presented in Supplementary Material S4A.

#### Faceted search examples

**Figure 8.**
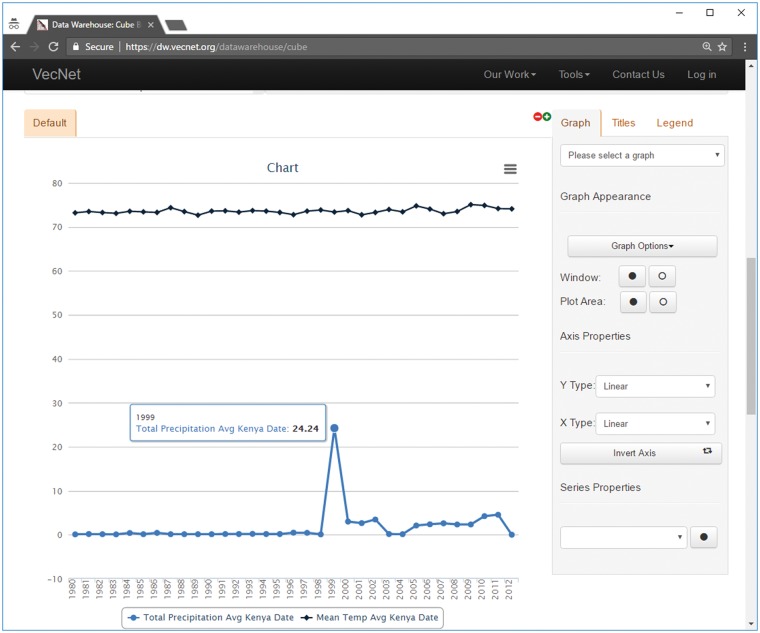
Screenshot of a faceted search with aggregated data on the Weather data cube. In this example, mean temperature and total precipitation data are aggregated over time by year, using the hierarchical ‘date’ dimension. The location Kenya → Nyanza → Kisumu is selected from the hierarchical dimension ‘location’, using the ‘Data Slicer’ panel. Various properties of the generated graphs (type, title, legend, axes, series etc.) can also be modified. The tooltip, as shown in the figure, appears when hovering over a data point in a data series, showing the value (24.24 in this example) of the data point and the name of the data series.

**Figure 9.**
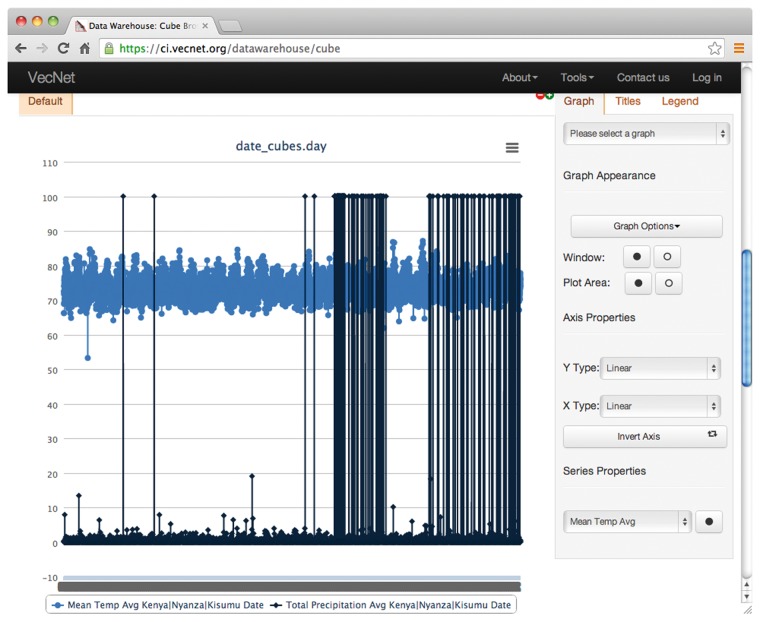
Screenshot of graphs generated from non-aggregated data. This example shows the resulting graphs of a faceted search with non-aggregated data on the Weather data cube. Temperature and precipitation data are displayed over time by day, using the hierarchical ‘date’ dimension. The location Kenya → Nyanza → Kisumu is selected from the hierarchical dimension ‘location’, using the ‘Data Slicer’ panel.

Faceted search allows both ‘aggregated’ and ‘non-aggregated’ forms of historical data to be accessed using the interface. Users can view the results from the faceted searches in multiple forms (e.g. graphical or tabular), and also download them for offline analysis. The general workflows for both forms are similar, and described below through examples in Figures 8 and 9.

Figure 8 shows an example of the resulting graphs of a faceted search with ‘aggregated’ data for the Weather data cube, where average temperature and total precipitation data are aggregated over time ‘by year’ for the entire dataset (1980–2012) using the hierarchical ‘date’ dimension. The location Kenya → Nyanza → Kisumu is selected from the hierarchical dimension ‘location’, using the ‘Data Slicer’ panel. Various properties of the generated graphs (type, title, legend, axes, series etc.) can also be modified. The tooltip, as shown in the figure, appears when hovering over a data point in a data series, showing the values (24.24 in this example) of the data point and the name of the data series. The tabular view, which can be printed or downloaded in various formats (including plain text, spreadsheet and PDF), is presented in Supplementary Material S4C.

‘Non-aggregated’ data may be considered as a special case of aggregated data (data aggregated at its ‘lowest level of granularity’). Continuing with the above example, the non-aggregated results, based on the temporal dimension ‘date’, consist of ‘daily’ values (instead of ‘yearly’ aggregates), which can be specified in the ‘Dimensions’ panel. Figure 9 shows the non-aggregated result graphs for the Weather data cube, where average temperature and precipitation are to be displayed over time by day (using the hierarchical date dimension) for the same location. The tabular view is presented in Supplementary Material S4D.

**Figure 10.**
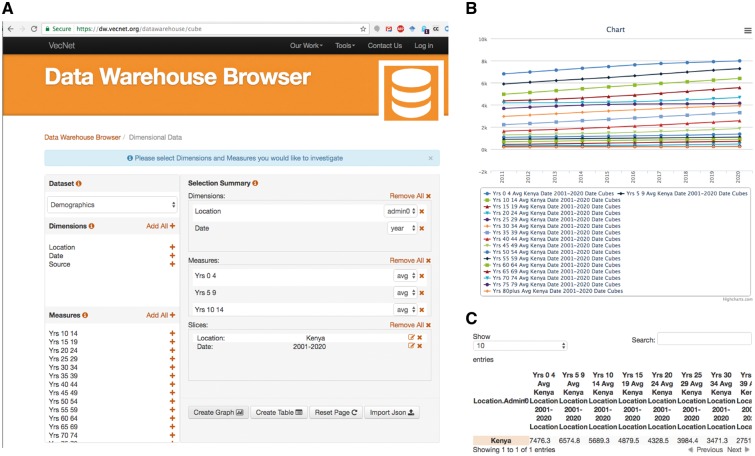
Use case example. A researcher wishes to investigate the effectiveness of a proposed new mosquito control intervention in a particular malaria endemic location. VecNet’s stored model EMOD (available via the Transmission Simulator, 24) will be used to model the current transmission baseline and to estimate the impact of various levels of coverage of the proposed new intervention. As part of the simulation setup, a population profile must be selected, specifying the distribution of the population over 5-year age intervals. In the Dimensional Data browser, the data cube Demographics is selected, along with two dimensions ‘location’ and ‘date’, and all age measures (i.e. years 0–4, 5–9 through 80plus). (A) Two data slices are specified, Kenya as the ‘location’ and 2001–20 as the ‘date’. **(B)** The resulting age distribution graph of this query. **(C)** The resulting table of this query, which is then used to either build a custom profile or select from an option provided by the EMOD web interface.

We present a typical use case (Figure 10) of the ‘Dimensional Data’ browser as follows: a researcher wishes to investigate the effectiveness of a proposed new mosquito control intervention in a particular malaria endemic location. At the *VecNet* online portal, the simulation model EMOD (available via the Transmission Simulator, 36) will be used to model the current transmission baseline and to estimate the impact of various levels of coverage using the proposed new intervention. As part of the simulation setup, a population profile must be selected from several profiles provided, specifying the distribution of the population over 5-year age intervals, 0–4, 5–9, 10–14 etc. In the DW browser, the data cube Demographics is selected, along with two dimensions ‘location’ and ‘date’, and all age measures (i.e. years 0–4, 5–9, through 80plus). Finally, two slices are specified, Kenya as the location and 2001–20 as the date, as shown in Figure 10A. The resulting age distribution graph and table of this query are shown in Figure 10B and Figure 10C, respectively. The table is then used to either build a custom profile or select from an option provided by the EMOD web interface.

### The ‘lookup tables’ browser

The ‘Lookup Tables’ browser allows access to all lookup tables, which serve as auxiliary tables to hold static data. Mostly, these records are textual and non-numeric in nature, and cannot be meaningfully aggregated using the dimensions. This type of data is used primarily to help users to parameterize different models, and to form guided opinions about a variety of topics. Given its textual nature, the data is most suitable to be presented only in tabular formats. A separate interface for the non-numeric data allows a better presentation to the users as well as to other models of VecNet. It also ensures that accidental aggregations over non-numeric values can be prevented.

**Figure 11.**
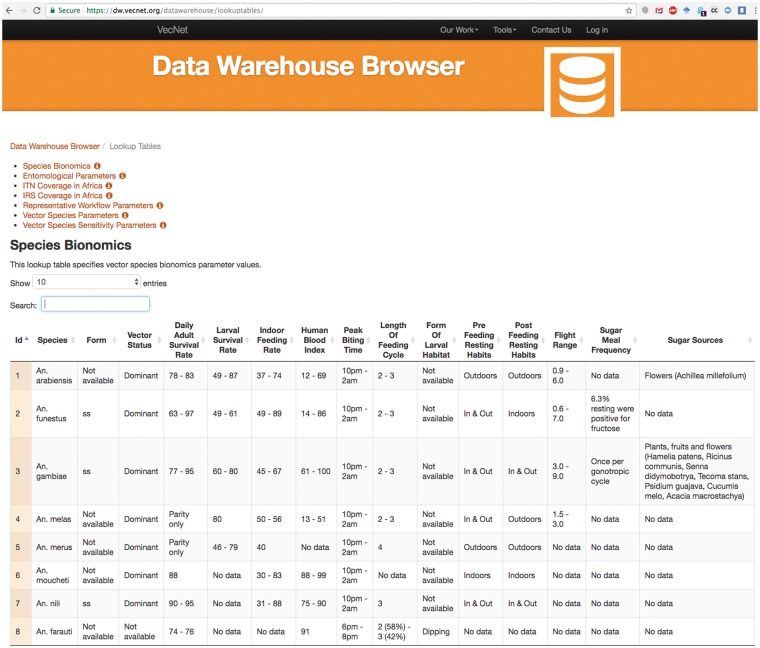
Screenshot of the lookup tables browser example. The screenshot depicts a partial view of the Species Bionomics lookup table, which stores bionomics parameter values for different mosquito species. Users can select or deselect all or any number of parameters (columns), and sort the tabular view by any parameter. The selected data can be copied as plain text, or downloaded in different formats (CSV, PDF etc.).

Figure 11 depicts a screenshot of the Species Bionomics lookup table, listing several bionomics parameter values for different mosquito species (e.g. form of larval habitat, flight range, indoor feeding rate etc.). The data can be copied as plain text, or downloaded in different formats (CSV, PDF, etc.). The ‘Search’ input box provides live search functionality for all existing parameters, and works as a live filter. For example, typing the characters ‘fun’ in the ‘Search’ box filters the table with a single record of *An. funestus*.

### The ‘results viewer’

The ‘Results Viewer’ interface, available through the Transmission Simulator (36), allows users to navigate through the simulation-based predictive data, which are generated as outputs of the stored simulation models (EMOD and OpenMalaria). The ‘scenario-based’ visualization interface allows users to graphically view and analyse the stored data series results from a specific Transmission Simulator run, and is useful for comparing data from within the same scenario or run of a simulation. On the other hand, the ‘custom interface’ is provided to visualize data simultaneously (i.e. on the same chart or graph) from multiple Transmission Simulator runs, which allows users to create custom charts that are not bound by simulation model, scenario, or homogenous output types. This, in turn, allows users to access the results of stored model parameterizations, or a public set of parameterizations, and perform a variety of quantitative tasks including trend analysis, outlier identification etc.

**Figure 12.**
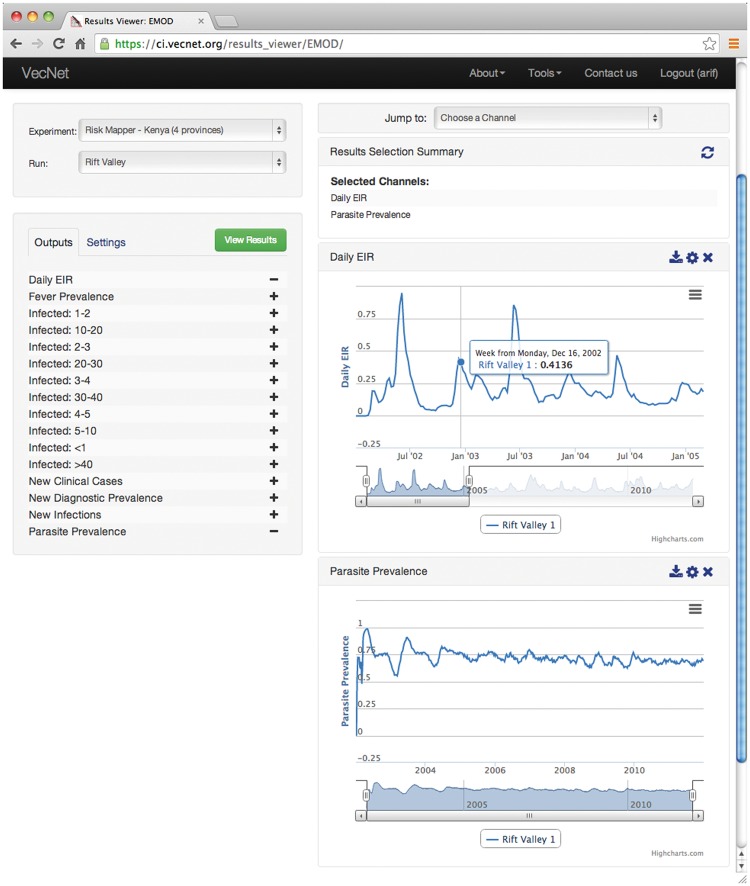
Screenshot of the ‘Results Viewer’. The ‘Results Viewer’ interface is available for public viewing through the Transmission Simulator (24). In this example, output channels ‘Daily EIR’ and ‘Parasite Prevalence’ are selected to be displayed from a ‘Rift Valley’ run from the ‘Risk Mapper—Kenya’ experiment. The resulting graphs are displayed on the right. For each graph, the user can zoom in using the slider placed under the x-axis, by dragging out a rectangle in the graph. In this example, the ‘Daily EIR’ graph is zoomed in to display a monthly view of the data series (from July 2002 to January 2005).

Figure 12 shows an example of navigating the simulation-based predictive data, in which output channels ‘Daily EIR’ and ‘Parasite Prevalence’ are selected to be displayed from a ‘Rift Valley’ run from the ‘Risk Mapper—Kenya’ experiment. The resulting graphs are displayed on the right. For each graph, the user can zoom in using the slider placed under the x-axis, by dragging out a rectangle in the graph. In this example, the ‘Daily EIR’ graph is zoomed in to display a monthly view of the data series (from July 2002 to January 2005). Another example is given in Supplementary Material S4E.

We envisage that eventually this interface will provide faceted search functionalities for all dimensions of the snowflake schema of the simulation-based predictive data (see Figure 3). However, even at its current stage, it offers valuable capabilities to the users, helping them to retrieve important parameter sets for visual or offline analysis.

## Discussions

Some of the key features, characteristics and future directions of VecNet-DW are highlighted below.

### Design methodology

To facilitate rapid development, we use a ‘bottom-up’ approach (as opposed to a time-consuming ‘top-down’ approach, which requires a global enterprise model) (9, 11) and a ‘centralized’ architecture (as opposed to ‘federated’ or ‘tiered’ architectures), which stores all data from various data marts into a ‘single’ DW (9). Initially, we follow the ‘source-driven’ (as opposed to ‘analysis-driven’) approach for the logical/conceptual design (8). This approach allows us to analyse and collect information from the heterogeneous sources to the DW. However, as the DW matures, the analysis-driven approach, which centres on the analysis requirements of the users, would be combined with the source-driven approach into an iterative development methodology. This, in turn, would ensure the correspondence of the analysis requirements with the available information in the DW.

Because of its ability to yield rapid turnarounds on deliverables, the ‘iterative and incremental’ development model is advocated as a key to success for a DW implementation (45). As we progressively advance the DW by incorporating new sources of data, many of the development steps are undertaken in a series of iterative refinements. Adjustments and enhancements of features to the data sets in previous iterations are made based on the collected feedback. Since the users ultimately decide on the success of any DW (45), we consider the direct involvement of our end-users in the early development phases as a critical success factor.

### Open-source software and open data access policy

As part of the VecNet Cyberinfrastructure (CI), *VecNet-DW* uses open-source software components for its various elements. All software used are freely available, mature, and widely used, and provide a low risk, maintainable, and portable design. The contents of *VecNet-DW* are partly ‘open access’, allowing users to have unrestricted ‘data’ access (the ‘compute’ access, however, is limited to only ‘expert users’). As non-public data is also stored within *VecNet-DW*, we implement privacy services, ensuring the protection of information being transmitted from and to the DW. For example, to protect users’ privacy, only a selected subset of predictive data is made available for public viewing through the Results Viewer interface via the Transmission Simulator (36).

### Star vs. Snowflake schemas

As discussed before, in *VecNet-DW*, the historical and predictive data are modelled using the star (or, constellation) and snowflake schemas, respectively. Since each schema type has its own benefits and drawbacks, a case-by-case analysis, based on metrics and features of the desired characteristics of the DW, is used to determine the suitable type for the two data categories, as described in Table 3. The star schema becomes a suitable choice for historical data for the highly structured nature of the data and its low degree of normalization. On the other hand, the enormous volume and less structured nature of predictive data make the snowflake schema a more natural choice.

**Table 3.** Comparison of star and snowflake schemas for historical and predictive data, respectively

Feature/Metric	Star schema	Snowflake schema
Type of modelled data	historical	predictive
Data modelling approach	top-down	bottom-up
Structure of data	highly structured	less structured
Degree of normalization	low or moderate	high
Query execution time	less	more
Number of joins per aggregation	fewer	more
Speed of aggregation	faster	slower
Update on records	more complicated	simpler
Disk storage requirements	higher	lower
Number of conformed dimensions across data marts	many	few
Average data volume per table	<100 million rows	>100 million rows
Ease of maintenance	more complicated	easier

In *VecNet-DW*, historical data are modelled using the star schema, while predictive data are modelled using the snowflake schema. The ‘star’ schema is used for historical data due to its advantages of faster aggregation speed, shorter response times, etc. On the other hand, the ‘snowflake’ schema becomes a more natural choice for predictive data due to its lower disk storage requirements and ease of maintenance.

### Performance aspects

Currently, response times for complex queries in the DW are in the range of milliseconds. For example, for a complex weather query, the response time (measured using a feature in the Google Chrome browser) is 500 ms.

Due to the enormous volume of predictive data (larger than 100 million rows), the high disk storage requirement soon becomes a concern for the DW. As the number of rows grows, a given row in the fact table causes significant increase in the normalized dimension tables. Trying to minimize the disk storage requirement would cause more complex queries with higher number of joins, which, in turn, would result slower aggregation speed.

To alleviate this, the stored predictive data (from simulation models) are partitioned ‘by simulation’, allowing us to restrict the number of rows needed to be searched and joined for any given query to be <100 million rows. Data partitioning increases the average query execution speed for aggregations, and, more importantly, also sets a threshold on query execution times. This ensures that any given query will require searching on a ‘single’ partition of the stored data, and the query execution time would be bounded by (no more than) the time required to read the largest partition on the server (assuming all other factors remain unchanged). The partitioned fact tables allow only a small subset of data to be searched against a query, facilitating high availability and low query times for *VecNet-DW*. The maintenance operations related to data partitioning are performed on the simulation results on a monthly basis. Thus, for predictive data, the use of data partitioning allows us to overcome the major drawbacks of the snowflake schema: the DW achieves faster speed for aggregation queries to a degree which is desired for most OLAP applications, while minimizing the disk storage requirements on the system by saving hundreds of gigabytes of storage. However, for historical data, the marginal gains in speed and storage, at a cost of significant losses in the ease of data updates and maintenance prohibit the use of data partitioning.

### Model-to-model comparison

The outputs from the two stored models (EMOD and OpenMalaria) contain little similarity, except for the format of ‘time series’ data as the output variables. This motivated us to dimensionally model and store the outputs in the DW in time series format (in the snowflake schema used for predictive data). This also allows the DW to retain the most detailed level of temporal granularity with which the outputs were originally produced by the two models.

However, as became apparent in the DW design phases, both models use different sets and combinations of input variables to generate various output variables, some of which share the ‘same’ names across the models (e.g. EIR). Since these variables may have subtle differences in their definitions, meanings, and calculation procedures, they may not be directly comparable across the models. Until further future work eliminates these differences, we store both lists of variables on a ‘per-model’ basis. This will be especially useful for expert and advanced users who understand both models comprehensively. 

### Dynamic integration and application programming interface

One of the design goals of *VecNet-DW* is its seamless integration capability with the other major components of VecNet CI (3), especially the Digital Library and the Integrated Modelling Platform. Since the primary components of *VecNet-DW* are based on the Python programming language (46), it integrates well with a wide variety of other programming languages and tool-sets. The two Python-based Web application frameworks, ‘cubes’ (41) and Django (42), provide the flexibility to create dynamically generated interfaces based on the preferences and roles of a set of given users. They also facilitate dynamic generation of and operations on the OLAP data cubes, making these query-able through VecNet’s Digital Library (24) and other third party software components/tools.

To meet this end, we equip *VecNet-DW* with an application programming interface (API), which allows individual users and other third party tools to browse, aggregate, and download portions of *VecNet-DW* content for offline analysis. The JSON-enabled Web API provides direct links (Web addresses) for all browsable data at the bottom of the pages, generated during the faceted searches in *VecNet-DW* Web Portal. This is especially helpful to mitigate the limitations of the Portal in cases when its existing analysis tools may seem inadequate for a given task. In the future, we plan to extend the API by writing and distributing drivers for direct use with a variety of analysis tools.

### Miscellaneous issues

Modelling the three categories of data with its most detailed level of granularity in *VecNet-DW* allows the users to select from aggregated as well as non-aggregated forms of searches. In the modelling process, expert users (e.g. modellers) can visually interpret, analyse, and decide whether to accept or reject the data, before they actually submit simulation jobs in VecNet.

In some cases, the star or constellation schema, which is used to model the historical data, may not represent the best design technique. As we strive to achieve an appropriate balance between traditional databases and DM techniques, in future, these situations would be handled by considering our DW needs as well as the performance criteria, rather than reliance upon specific technical panaceas (e.g. the star schema).

In most cases, unlike other biological DW projects [e.g. BioMart (12–15), transformation of existing content from third normal form into a DM schema was not necessary for modelling historical or static data, because the bulk of the data sources we worked with so far were stored as flat files, spreadsheets etc. (e.g. non-RDBMS data sources). However, for query performance and optimization issues, we took the leverage of using an RDBMS as a back-end to our multi-dimensional schema.

### Future directions

As *VecNet-DW* experiences continuous improvement cycles, we will continue to follow the iterative development methodology, and progressively advance the DW by incorporating new data sources. We plan to add new data cubes related to other categories of malaria-related data such as geo-referenced EIR, rainfall, insecticides, and other malaria control interventions. We also plan to improve the faceted search techniques and the geo-spatial aspects of navigation (e.g. by including customized geographical maps for data browsing). To improve the historical data representation, the spatial and temporal dimensions are also likely to be re-modelled. The snowflake schema for modelling predictive data is also being updated to store and present the simulation results from the OpenMalaria model. General performance criteria of the DW (e.g. query response time) are also being continuously refined.

In addition, for all three major components of *VecNet-DW* (the ‘Dimensional Data’ browser, the ‘Lookup Tables’ browser and the ‘Results Viewer’), we also plan to enhance the suite of open-source tools used to process and analyse the DW’s content, with two major goals of: (i) providing better analysis capability, and (ii) decreasing the need of external tools for post-processing tasks. Significant security challenges also need to be addressed and overcome to allow third-party users’ access to *VecNet-DW* API. 

## Conclusions

*VecNet-DW* can translate stakeholder questions into appropriate analyses and visualizations of model predictions, providing a robust data environment for VecNet users. The power of the DW emerges from integrated querying of its different data marts, and by structuring those queries according to the desired dimensions.

## Supplementary data

Supplementary data are available at *Database* Online.

## Supplementary Material

Supplementary File1 Vocabulary

Supplementary File2 BusMatrix

Supplementary File3 Frameworks

Supplementary File4 AdditionalScreenshots
