# Supplementary material for: An online analytical processing multi-dimensional data warehouse for malaria data
Source: Database (Oxford). 2017 Oct 7;2017:bax073. doi: 10.1093/database/bax073 (PMC5632519; doi:10.1093/database/bax073)
Supplement: Supplementary File1 Vocabulary [file bax073_Supp_File1_Vocabulary.doc]

**Supplementary File 1 - Vocabulary of Relevant Data Warehouse Terms**

The following vocabulary describes relevant terms (in alphabetical order) used in the context of malaria-related data for *VecNet-DW*, with examples as necessary. The conventions used in this paper for different types of entities are described in Table 1.

- **Bus matrix:** a tabular tool used to create, document, and communicate all data marts at a glance; the rows of the bus matrix represent the data marts, and the columns represent the dimensions used across the DW; if a dimension (column) is related to a data mart (row), the corresponding cell in the bus matrix is marked accordingly. Example: see Supplementary File S2.
- **Conformed dimension:** a common, structured dimension that is shared across multiple fact tables; they are used to avoid redundant data in the DW. Examples: *date* and *location* in Figure 2.
- **Constellation schema:** also known as the *galaxy* schema, the constellation schema is a collection of the *star* schemas in the DW; it ties together all the fact tables, dimension tables, and lookup tables; it allows conformed dimensions and facts to be shared, thereby facilitating the realization of the DW enterprise bus architecture; however, the fact tables in the schema do not need to be directly related. Examples: see Figure 2. Also see: star schema, snowflake schema.
- **Data mart:** a logical and physical subset of the DW, usually representing data from a single process of interest; deciding the data marts, and the sources of data they would contain, is one of the crucial early steps in the design of the dimensional model (11); each data mart may contain one or more fact tables. Examples: Household Surveys, Artemisinin-based Combination Therapy (ACT), Indoor Residual Spraying (IRS), Insecticide-Treated Net (ITN), etc.
- **Definition:** the explanatory description of a term, which is stored in a dictionary. Example: *Human blood index* is the proportion of blood-fed mosquitoes that contains human blood.
- **Dictionary:** contains definitions of terms which need explanatory descriptions; the terms usually appear in lookup tables, which are related to the dictionary. Examples: *species parameters*, *entomological endpoints phases*, etc.
- **Dimension:** a descriptive attribute that provides context to the facts, and describes the “who, what, when, where, why, and how” about the facts (11); dimension attributes serve as the primary source of query constraints, groupings, and report labels; dimensions may be hierarchical and non-hierarchical. Examples: (i) hierarchical: *date* with hierarchy *year-month-day*, etc; (ii) non-hierarchical: *land use*, *chemical class*, etc.
- **Dimension table:** a table in the DM with a single-part primary key and descriptive (textual) attribute columns; as integral companions to a fact table, dimension tables serve as the entry points into the fact table; each dimension table may have many columns or attributes, which describe its rows. Examples: *location*, *date*, etc.
- **Fact:** a numeric, quantitative measure stored as part of a row in a fact table; the most useful facts are usually numeric and additive. Examples: total population who slept under an ITN, proportion of households with at least one bednet, etc. in the fact table Household Surveys.
- **Fact table:** a primary table in the dimensional model (DM) to store the facts; each fact is characterized by a composite key, composed of foreign keys (a foreign key is a field in a dimension table that uniquely identifies a row of another table; a composite key may consist of multiple foreign keys) drawn from the dimension tables. Thus, the keys are used to link the fact and dimension table. Examples: Household Surveys, IRS Operational Coverages, etc.
- **Lookup table:** also known as *reference tables*, contain unordered collection of values (mostly textual) that are stored in relational tables (in the RDBMS); each lookup table is an auxiliary table to hold static data, and is used to lookup values, which may be related to individual dictionaries; sometimes, it can also be used to translate an encoded/obscure epidemiology term into an explanatory description for reporting purposes; lookup tables save space, improve flexibility, and allow the DW to describe a coded value to change while retaining its meaning. Examples: Species Bionomics, Entomological Parameters, etc.
- **Record:** a generic term used to describe a row in the fact tables, dimension tables, and lookup tables.
- **Snowflake schema:** a logical arrangement of fact tables in a multidimensional DW such that the entity relationship diagram resembles a snowflake shape; it is represented by centralized fact tables which are connected to multiple dimension tables.
- **Star schema:** the simplest type of DW schema; each *star schema* consists of one or more fact tables referencing several dimension tables; the star schema is an important special case of the snowflake schema, and is more effective for handling simpler queries.
