# Supplementary material for: An online analytical processing multi-dimensional data warehouse for malaria data
Source: Database (Oxford). 2017 Oct 7;2017:bax073. doi: 10.1093/database/bax073 (PMC5632519; doi:10.1093/database/bax073)
Supplement: Supplementary File2 BusMatrix [file bax073_Supp_File2_BusMatrix.doc]

**Supplementary File 2 - Bus Matrix for the DW**

| **Data Mart** | *location* | *date* | *source* | *subgroup* |
| --- | --- | --- | --- | --- |
| Demographics | X | X | X |  |
| Gross National Income | X | X |  |  |
| Household Surveys | X | X | X | X |
| Households | X | X | X | X |
| Artemisinin-based Combination Therapy (ACT) | X | X |  |  |
| Indoor Residual Spraying (IRS) | X | X |  |  |
| Insecticide-Treated Net (ITN) | X | X |  |  |
| Weather | X | X |  |  |

**Bus Matrix for the Data Warehouse.** In the bus matrix, an `X' in a cell represents the correspondence of the dimension (column) with the data mart (row). Separate data marts can be implemented by different development groups at different times, and can be plugged together and usefully coexist. The bus matrix is independent of the technology and the database platform. The coherent data marts share a uniform architecture of *conformed* dimensions and facts.
