# Supplementary figures and images for: An online analytical processing multi-dimensional data warehouse for malaria data
Source: Database (Oxford). 2017 Oct 7;2017:bax073. doi: 10.1093/database/bax073 (PMC5632519; doi:10.1093/database/bax073)

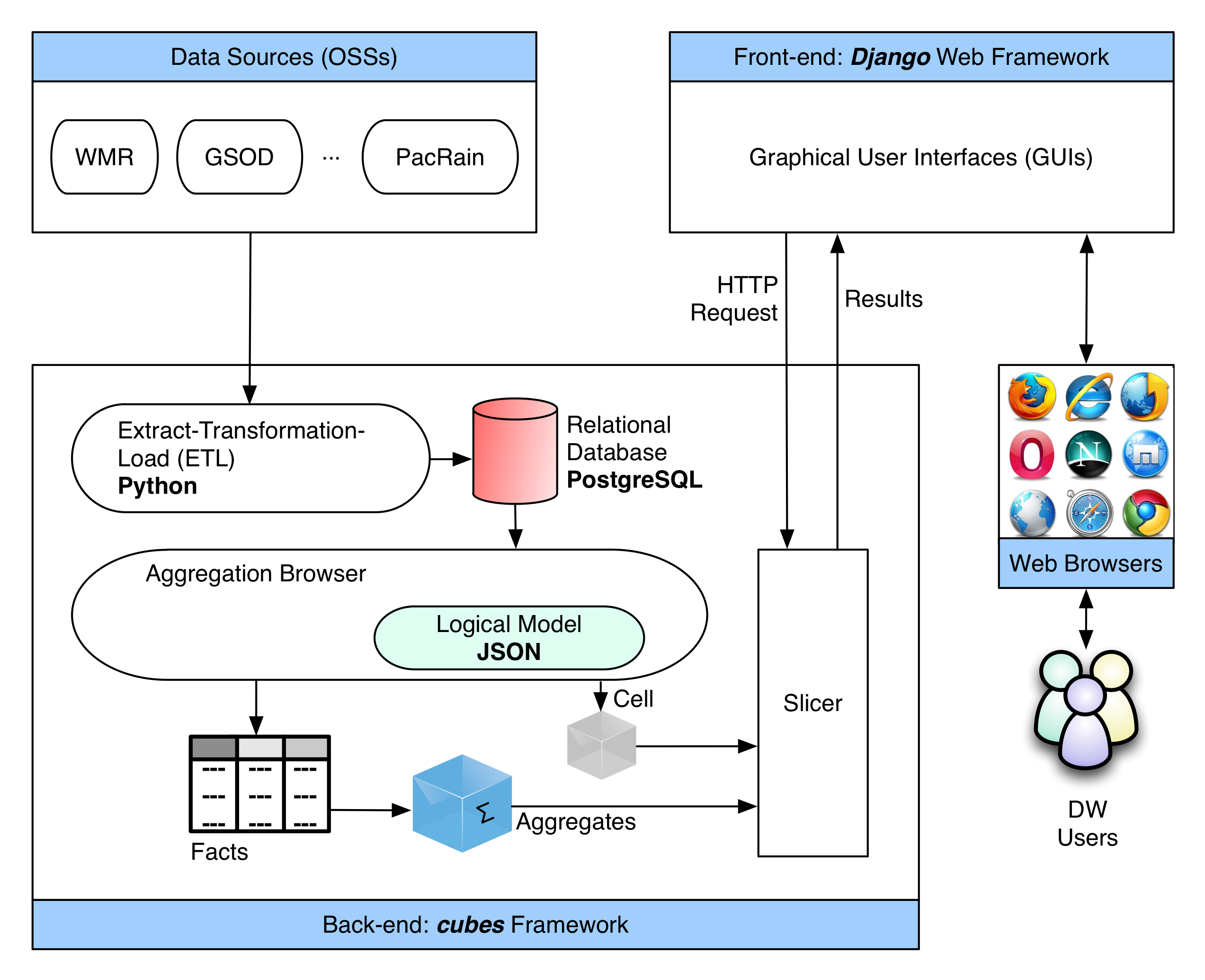

Supplement: Supplementary File3 Frameworks [file bax073_Supp_File3_Frameworks.png]
