# Supplementary material for: An online analytical processing multi-dimensional data warehouse for malaria data
Source: Database (Oxford). 2017 Oct 7;2017:bax073. doi: 10.1093/database/bax073 (PMC5632519; doi:10.1093/database/bax073)
Supplement: Supplementary File4 AdditionalScreenshots [file bax073_Supp_File4_AdditionalScreenshots.doc]

**Supplementary File 4 - Additional Screenshots**


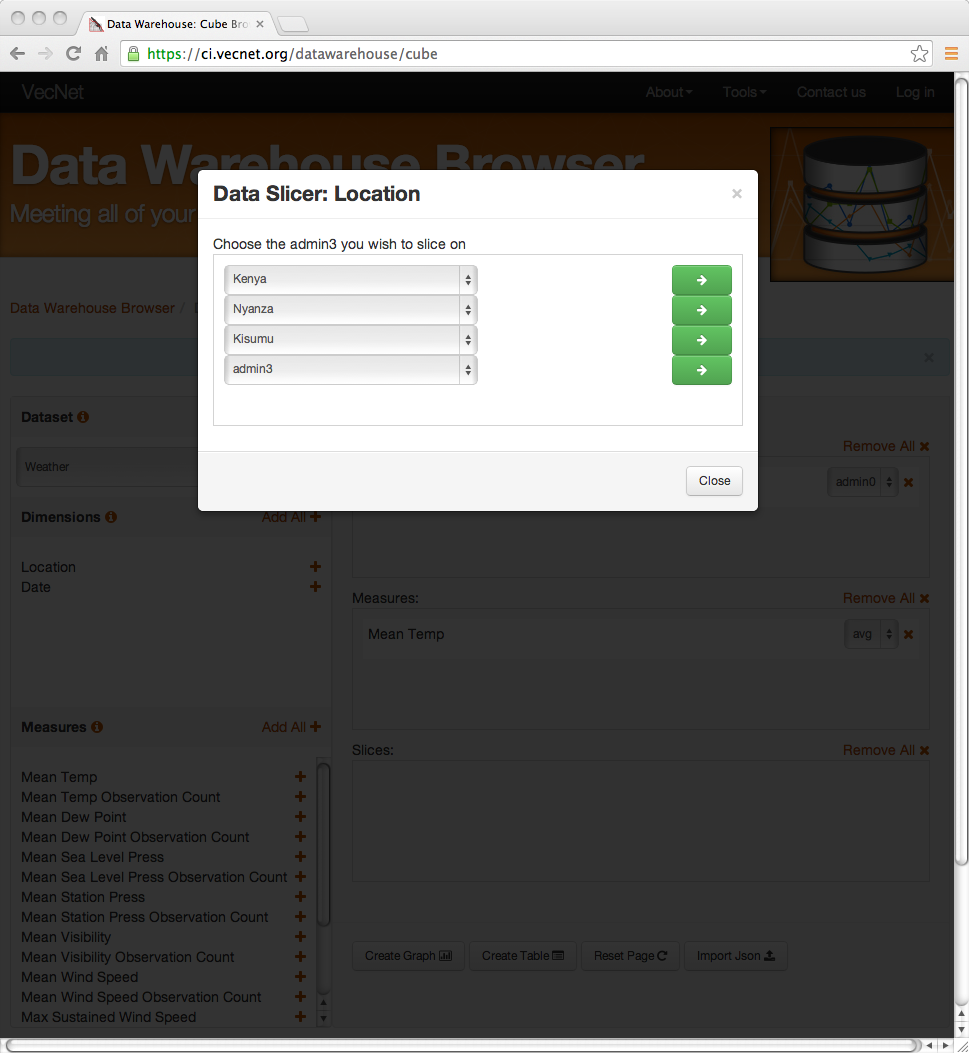


A. Screenshot of the Data Slicer Panel in the Dimensional Data Browser


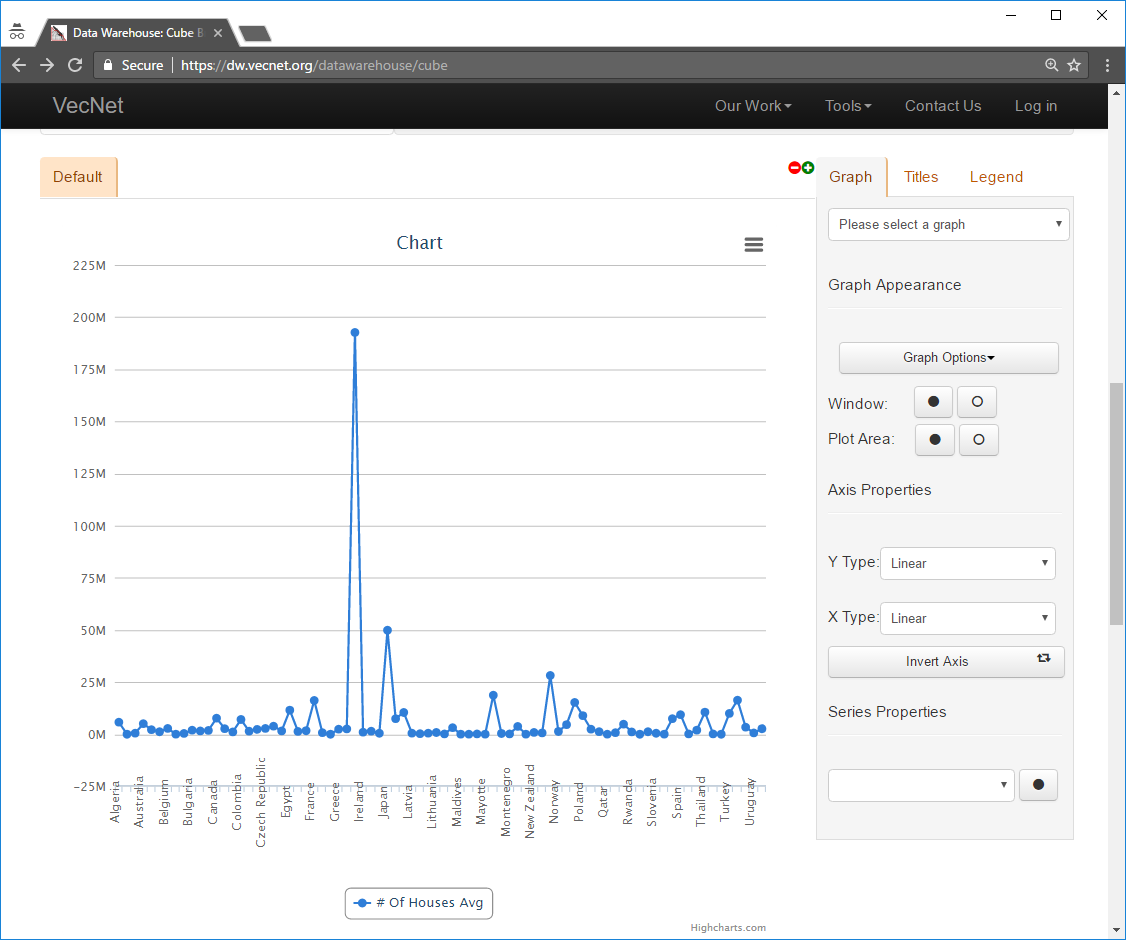


B. Screenshot of axis thinning. When there are too many items on the X-axis to display, some of them will be skipped in regular intervals. This screenshot shows the resulting graph of a query on the Households cube. Labels of more than 100 countries are ‘thinned’ along the X-axis as they do not fit on a single screen. A user can still hover over the chart to get intermediate values (country name in this example).


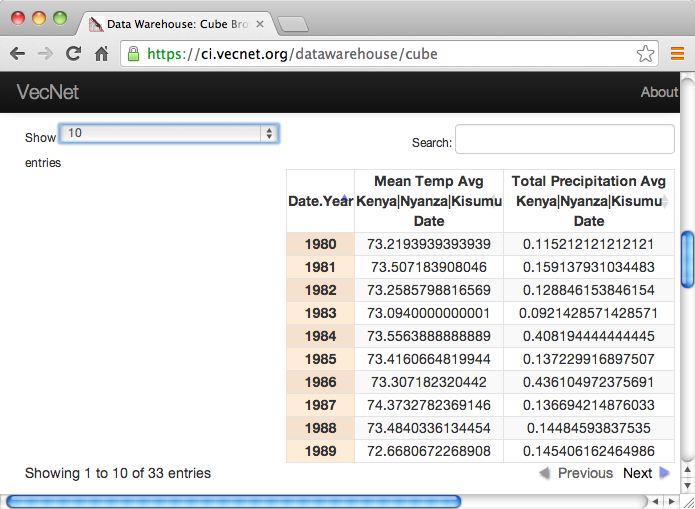


C. Screenshot of an Example of Tabular View Generated from Aggregated Data


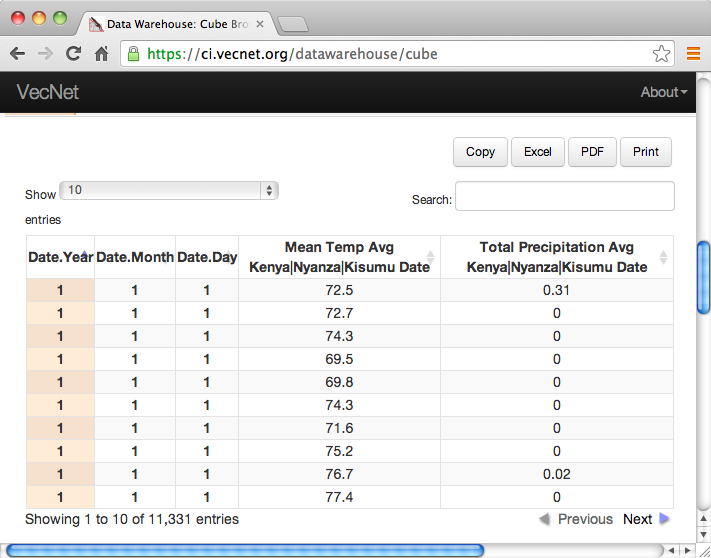


D. Screenshot of an Example of Tabular View Generated from Non-Aggregated Data


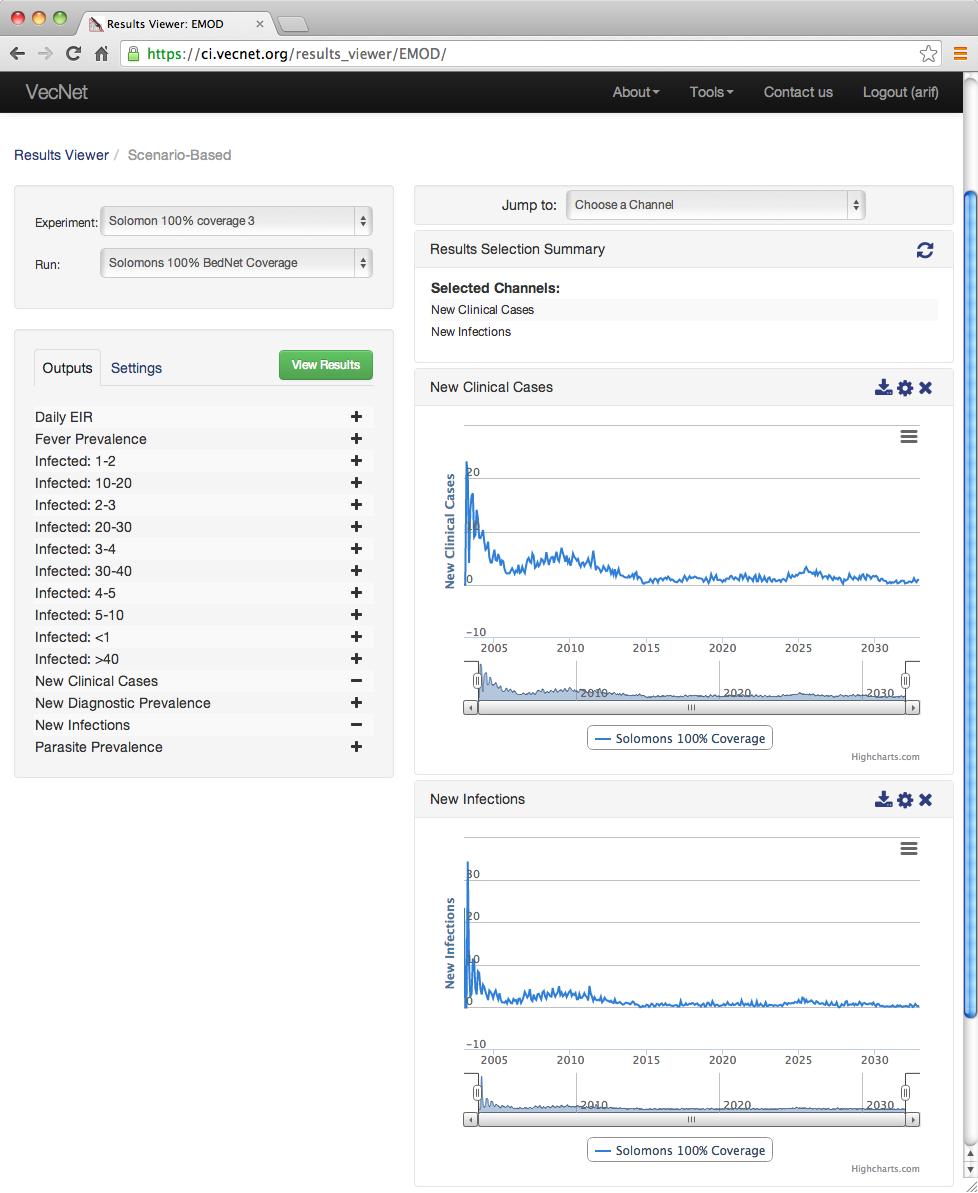


E. Screenshot of an Example of the Results Viewer Interface with “Solomon Islands”
